# Bortezomib-resistance is associated with increased levels of proteasome subunits and apoptosis-avoidance

**DOI:** 10.18632/oncotarget.12731

**Published:** 2016-10-18

**Authors:** Yi-Xin Wu, Jia-Hua Yang, Hirotomo Saitsu

**Affiliations:** ^1^ Department of Biochemistry, Hamamatsu University School of Medicine, Hamamatsu, Japan; ^2^ Department of General Surgery, Putuo Hospital, Shanghai University of Traditional Chinese Medicine, Shanghai, China

**Keywords:** bortezomib resistant, proteasome inhibitor, hepatocellular carcinoma cell, BCL2, apoptosis

## Abstract

Bortezomib (BTZ), a proteasome inhibitor, is the first proteasome inhibitor to be used in clinical practice. Here we investigated the mechanisms underlying acquired bortezomib resistance in hepatocellular carcinoma (HCC) cells. Using stepwise selection, we established two acquired bortezomib-resistant HCC cell lines, a bortezomib-resistant HepG2 cell line (HepG2/BTZ) and bortezomib-resistant HuH7 cell line (HuH7/BTZ). The 50% inhibitory concentration values of HepG2/BTZ and HuH7/BTZ were respectively 15- and 39-fold higher than those of parental cell lines. Sequence analysis of the bortezomib-binding pocket in the β5-subunit showed no mutation. However, bortezomib-resistant HCC cells had increased expression of β1 and β5 proteasome subunits. These alterations of proteasome expression were accompanied by a weak degree of proteasome inhibition in bortezomib-resistant cells than that in wild-type cells after bortezomib exposure. Furthermore, bortezomib-resistant HCC cells acquired resistance to apoptosis. Bortezomib up-regulated pro-apoptotic proteins of the Bcl-2 protein family, Bax and Noxa in wild-type HCC cells. However, in bortezomib-resistant HCC cells, resistance to apoptosis was accompanied by loss of the ability to stabilize and accumulate these proteins. Thus, increased expression and increased activity of proteasomes constitute an adaptive and auto regulatory feedback mechanism to allow cells to survive exposure bortezomib.

## INTRODUCTION

Hepatocellular carcinoma (HCC) is the most common primary liver cancer. The annual number of new HCC cases is more than one million, making it the fifth most common cancer worldwide and the third leading cause of cancer-related death [1, 2]. Surgery is currently the most effective treatment. However, it is only possible in a few patients (around 40%) [3]. Thus pharmacotherapy is considered to be the final and main treatment option for patients with advanced HCC. Regrettably, existing traditional chemotherapeutics have significant side effects. Moreover, the most patients with HCC have impaired liver function; aggressive medical therapy cannot be applied. Therefore, no effective treatment can be provided to these patients. Accordingly, there is need to develop novel therapies for HCC. In order to overcome these unsatisfactory aspects, several studies have been performed to elucidate the molecular mechanisms underlying HCC development and progression to identify targets for HCC treatment [4].

Recent studies have been shown that the activities of proteasome in tumor cells are higher than in normal cells. Furthermore, a number of proteasome substrates which are involved in cell cycle or apoptosis have been identified [5]. In many types of tumor cells, inhibition of proteasome activity leads to the accumulation several target proteins, such as IkB-α, Bax and p27, and subsequent induction cell cycle arrest or of apoptosis [6]. Therefore, proteasome inhibitors have been focused as a potential anticancer drug. Bortezomib, a proteasome inhibitor, is the first as an anti-cancer drug to clinical application, has been used in treatment of non-Hodgkin's lymphoma and multiple myeloma [7, 8]. Preclinical and clinical trials in both hematological malignancies and solid tumors have demonstrated that bortezomib is a relatively well-tolerated and active agent either as a single agent or in combination with traditional chemotherapeutic drugs [9–12]. The results of clinical trials of bortezomib in solid tumors suggest that the molecular targets of bortezomib in solid tumors are different from those reported in hematological malignancies. A phase I/II trial of bortezomib in patients with unresectable HCC has shown that bortezomib appears to be well tolerated in HCC patients [13, 14].

In many cases, bortezomib treatment develops rapidly in drug resistance. It is unfortunate that the mechanisms of resistance to bortezomib are poorly understood. Therefore, to develop the novel approaches to overcome bortezomib resistance, there is necessary to understand the mechanisms underlying this resistance. Recently, some mechanisms of proteasome resistance have been determined. Studies have showed that the bortezomib resistance in lymphoma and leukemia cell lines is due to mutations in the β5-subunit and β5 proteasome subunit overexpression [15–17]. One possibility is upregulation of the pathways that suppress apoptosis [18–24]. An association has been reported between overexpression of heat shock protein (HSP) 27 and bortezomib resistance in lymphoma cells [25, 26]. However, the mechanisms of bortezomib resistance in liver cancer are not yet clear.

To investigate the possible mechanisms of resistance to bortezomib in liver cancer, we established acquired bortezomib-resistant HCC cell lines, HepG2/BTZ and HuH7/BTZ. We examined the differential effects of bortezomib in wild-type and bortezomib-resistant HCC cell lines and found acquired resistance to apoptosis in these resistant cells. The cause of the resistance was due to loss of the ability to stabilize and accumulate pro-apoptotic proteins.

## RESULTS

### Acquired resistance to bortezomib in HepG2/BTZ and HuH7/BTZ cells

**Figure 1:**
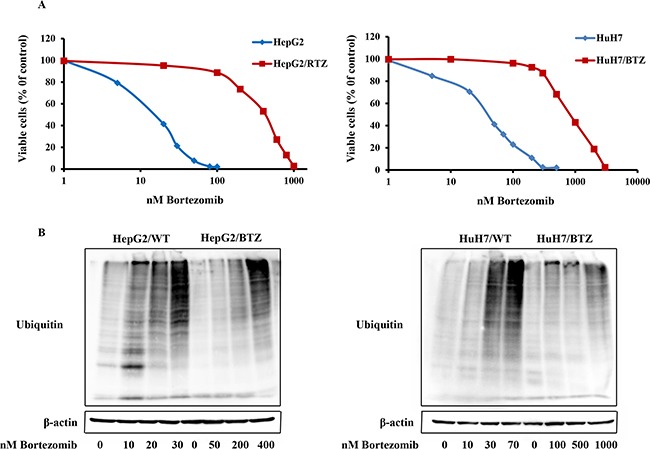
Resistance to the proteasome inhibitor bortezomib in bortezomib-resistant HCC cell lines HepG2/RTZ and HuH7/RTZ **A.** Dose-response curve of bortezomib-induced growth inhibition of wild-type HepG2 cells and bortezomib-resistant HepG2/RTZ cells. After treatment with various concentrations of bortezomib for 72 h, cell viability was measured using MTT assays. **B.** Bortezomib-resistant HCC cells demonstrate diminished accumulation of ubiquitinated proteins. Bortezomib-resistant HCC cells and their parental cells were treated with various concentrations of bortezomib for 72 h. Western blot analysis was performed using a monoclonal antibody against polyubiquitinated proteins.

Two bortezomib-resistant HCC cell lines, HepG2/BTZ and HuH7/BTZ, and their wild-type cells were examined for growth inhibition by bortezomib. Growth inhibition dose-response curves of bortezomib-induced cell showed 15-fold (IC_50_: 235 nM) and 39-fold (IC_50_: 1178 nM) levels of resistance in HepG2/BTZ and HuH7/BTZ cells, respectively, compared with those in their wild-type cells, wild-type HepG2 (IC_50_ 15.6nM) and wild-type HuH7 (IC_50_ 30.3 nM) cells after 72-h of treatment (Figure 1A and Table 1). In addition, HepG2/BTZ and HuH7/BTZ cells revealed cross-resistance to another proteasome inhibitor, MG132, but not to doxorubicin (Table 1).

**Table 1:** The IC_50_ values for each drug in HepG2 /BTZ, HuH7 /BTZ and their parental cell lines. The ratio of IC_50_ value of resistant to parental cell line is shown on the right

Drug	IC_50_(nM)		IC_50_ ratio	IC_50_ (nM)		IC_50_ ratio
	HepG2	HepG2/RTZ		HuH7	HuH7/RTZ	
Bortezomib	14.7	221	15.03	30.03	1178	38.9
MG132	370	2700	7.3	350	3100	8.9
Doxorubicin	205	307	1.48	1112	1902	1.71

### Bortezomib-resistant HCC cells are lost the ability to accumulate polyubiquitinated proteins in response to bortezomib

Bortezomib induced inhibition of protein degradation has accompanied accumulation of polyubiquitinated proteins. To investigate alteration of the protein degradation response to bortezomib in resistant HCC cells, polyubiquitinated proteins in bortezomib-resistant and wild-type HCC cells treatment with bortezomib for 72 h were checked using western blot analysis. Consistently, increases of polyubiquitinated proteins were observed in wild-type HCC cells but not in bortezomib-resistant cells at their selective concentration of 10−50 nM bortezomib (Figure 1B). However, the bortezomib-resistant cells showed accumulation of polyubiquitinated proteins upon exposure to bortezomib concentrations above the selective concentrations, >250 nM in HepG2/BTZ and >1000 nM in HuH7/RTZ (Figure 1B). Thus, bortezomib-resistant cells maintained their ability to accumulate ubiquitinated proteins, although this process occurred at higher bortezomib concentrations consistent with the resistance factor of bortezomib.

### Chymotrypsin-like and peptidylglutamyl-peptide hydrolyzing (PGPH) activities in bortezomib-resistant HCC cells are inhibited by much higher concentrations of bortezomib than in wild-type cells

**Figure 2:**
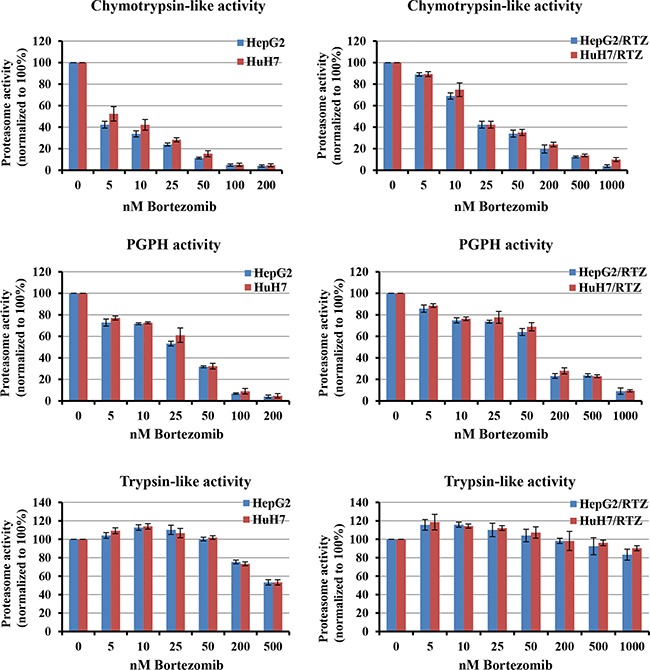
Bortezomib-induced inhibition of proteasome activities in parental and bortezomib-resistant HCC cells Chymotrypsin-, caspase- and trypsin-like activities were determined using intact cells after treatment with a concentration range of bortezomib for 24 h. Proteasome activities were normalized to untreated cells, which were set at 100%. Bortezomib-resistant cells (right panel) required higher bortezomib concentrations for proteasome inhibition comparable with the parental cells. All results represent the mean ± SEM of at least three experiments.

To characterize the activity of the ubiquitin-proteasome system in bortezomib-resistant HCC cell lines, we examined the inhibition of proteasome subunits activities in HepG2/BTZ, HuH7/BTZ cells and wild-type cells. In wild-type cells, after 24 h of treatment to bortezomib, levels of inhibition of chymotrypsin-like and PGPH activities were similar (Figure 2, left panel). For an 80% reduction in chymotrypsin-like and PGPH activities, concentrations ranging of bortezomib from 30 to 60 nM in wild-type HepG2 cells and 45 to 75 nM in wild-type HuH7 cells, respectively, were required. On the other hand, the trypsin-like activity was only slightly suppressed by a high bortezomib concentration of 200 nM. Compared to wild-type HCC cells, the bortezomib-resistant HCC cells required 3–4-fold higher bortezomib concentration to obtain similar inhibition of chymotrypsin-like and PGPH activities (Figure 2, right panel). Bortezomib did not inhibit the trypsin-like activity in HepG2/BTZ and HuH7/RTZ cells. These results demonstrated that after bortezomib exposure, the degree of proteasome inhibition is weaker in bortezomib-resistant HCC cells than in wild-type cells.

### Bortezomib-resistant HCC cells show changes in proteasome subunits activities due to increased expression of proteasome subunits

**Figure 3:**
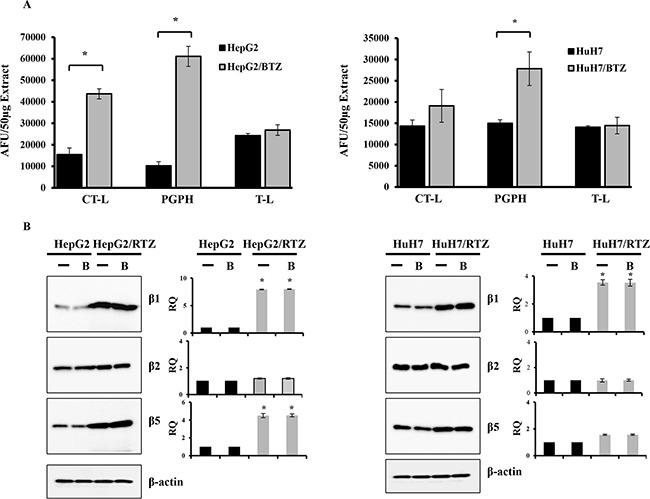
Bortezomib-resistant HCC cells show higher expression of constitutive proteasome subunits **A.** The three individual basal activities (CT-L, T-L and PGPH) of proteasomes in wild-type HCC cells were compared with those in bortezomib-resistant HCC cells. Proteasome extracts were prepared from HCC cells. The basal activities of CT-L, T-L, and PGPH were measured using specific fluorogenic peptide substrates for each activity. All results represent the mean ± SD of three independent experiments. *: P<0.01 as compared with the respective control. **B.** Western blot of proteasome subunits (β1, β2 and β5) and β-actin in wild-type HepG2, HepG2/BTZ, wild-type HuH7 and HuH7/BTZ cells treated with or without 30 nM bortezomib (B) for 24 h. RQ, relative quantity.

We next determined basal proteasome activities of bortezomib-resistant HCC cell lines in comparison with their parental cells. The basal activities of chymotrypsin-like and PGPH activities were 2.8- and 6-fold higher (P < 0.01) in HepG2/BTZ cells, and PGPH activity was 1.9-fold higher (P < 0.01) in HuH7/RTZ cells (Figure 3A). Statistical analysis showed a correlation between high basal chymotrypsin-like and PGPH activities and inherent bortezomib resistance (P < 0.01). Notable, no differences were detected in trypsin-like activity, means that the β2-subunit is not targeted by bortezomib. To investigate whether the increased proteasome subunit activities in bortezomib-resistant HCC cells might due to altered proteasome subunit expression, we determined the expression of proteasome subunit in HepG2/BTZ and HuH7/BTZ cells and their wild-type cells by western blot analyses (Figure 3B). Overall, higher expression of proteasome was seen in bortezomib-resistance cells compared to wild-type cells. The levels of β1 and β5 subunit expression were higher (P < 0.01) in HepG2/BTZ cells, and β2 subunit expression was higher (P < 0.01) in HuH7/RTZ cells (Figure 3B). These results demonstrate that the increased proteasome proteolytic activities in bortezomib-resistant HCC cells were caused by increased expression of proteasome subunits.

### Bortezomib-resistant HCC cells exhibit acquired resistance to apoptosis

**Figure 4:**
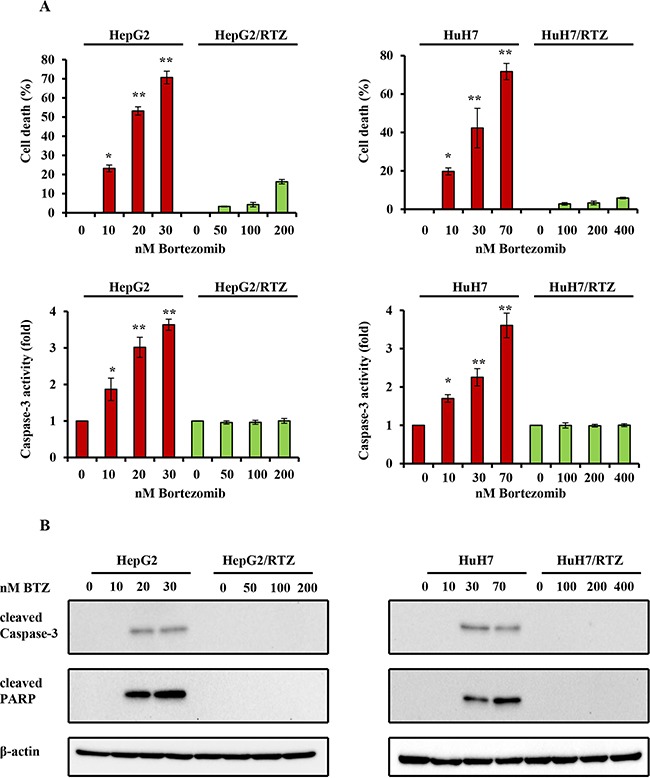
The dose-dependent analysis of caspase-3 activity and the expression of apoptosis-associated proteins in bortezomib-resistant HCC cells and their parental cells **A.** Wild-type HepG2, HepG2/BTZ, wild-type HuH7, and HuH7/BTZ cells were treated with various concentrations of bortezomib for 48 h. Apoptosis was then measured by cell viability and caspase-3 activity. **: *P*<0.01; *: *P*<0.05 as compared with the respective control. Data are the mean ± SD of three independent experiments. **B.** Wild-type HepG2, HepG2/BTZ, wild-type HuH7, and HuH7/BTZ cells were treated with various concentrations of bortezomib for 48 h. Expression levels of cleaved PARP, cleaved caspase-3, and β-actin (loading control) were evaluated by western blot analyses. BTZ, bortezomib.

**Figure 5:**
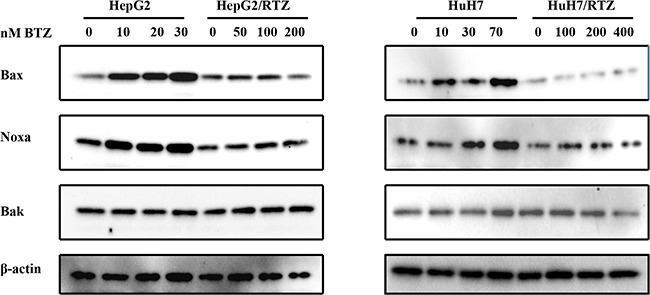
The dose-dependent analysis of Bcl-2 family proteins in bortezomib-resistant HCC cells and their parental cells Wild-type HepG2, HepG2/BTZ, wild-type HuH7, and HuH7/BTZ cells were treated with bortezomib at various concentrations for 48 h. Expression levels of Bax, Noxa, Bak, and β-actin (loading control) were evaluated by western blot analyses. BTZ, bortezomib.

It has been reported that bortezomib induce apoptosis in HCC cells. To confirm whether the resistance to bortezomib in HCC cells was correlated by apoptotic signaling, we examined the characteristics of resistant HCC cells, particularly apoptosis-regulating signals. As shown in Figure 4, resistant HCC cells exhibited resistance to apoptosis. Resistant HCC cells and their parental cells were treated with bortezomib for 48 h, and then cell viability and caspase-3 activity were measured. Caspase-3 activity was increased in all bortezomib treated wild-type cells in a dose-dependent manner. Compared with wild-type cells, bortezomib-induced caspase-3 activity and the percentage of cell death appeared to be lower in bortezomib-resistant HCC cells (Figure 4A). The expression of apoptosis-associated proteins was investigated in HepG2/BTZ, HuH7/BTZ cells and their wild-type cells. Activation of caspases-3 and cleavage of poly (ADP)-ribose polymerase (PARP) were investigated by western blotting after 48 h of exposure. As shown in Figure 4B, slightly increase in the activation of caspase-3 and subsequent cleavage of PARP after treatment with bortezomib in all wild-type HCC cells but not in bortezomib-resistant HCC cells. Additionally, we performed a time-dependent analysis of capase-3 activity, cell viability, and the expression of apoptosis-associated proteins in bortezomib-resistant HCC cells and wild-type cells. As shown in Figure 5, caspase-3 activity, the percentage of cell death, and the expression of apoptosis-associated proteins were increased in all bortezomib treated wild-type cells in a time-dependent manner. However, compared with wild-type cells, bortezomib-induced caspase-3 activity, the percentage of cell death, and the expression of apoptosis-associated proteins appeared to be failed to increase in response to in bortezomib-resistant HCC cells. These results demonstrated that the resistance to bortezomib in HCC cells was correlated by apoptotic signaling.

### Bortezomib-resistant HCC cells loss the ability to stabilize and accumulate pro-apoptotic proteins

**Figure 6:**
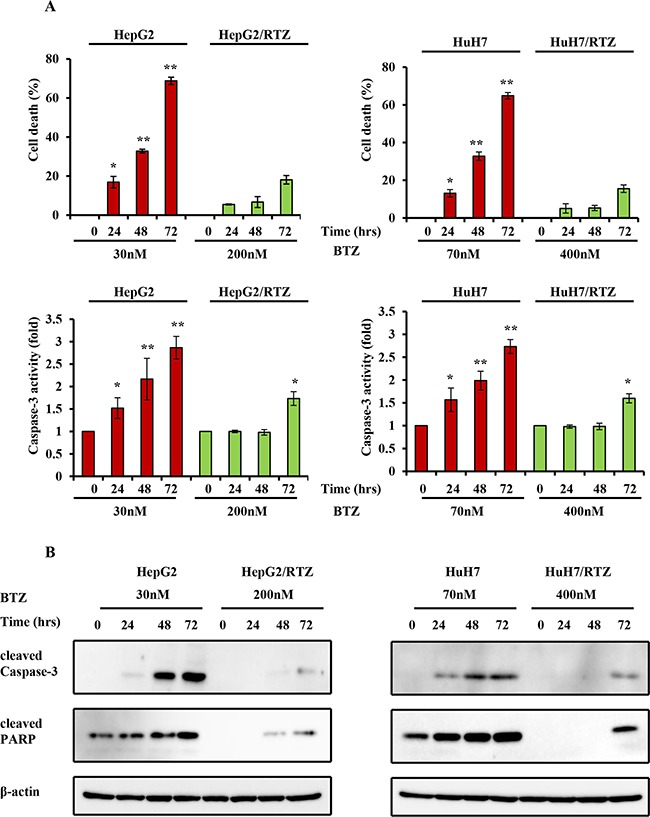
The time-dependent analysis of caspase-3 activity and the expression of apoptosis-associated proteins in bortezomib-resistant HCC cells and their parental cells **A.** Wild-type HepG2, HepG2/BTZ, wild-type HuH7, and HuH7/BTZ cells were treated with bortezomib for various times and then apoptosis was measured by cell viability and caspase-3 activity. **: *P*<0.01; *: *P*<0.05 as compared with the respective control. Data are the mean ± SD of three independent experiments. **B.** Wild-type HepG2, HepG2/BTZ, wild-type HuH7, and HuH7/BTZ cells were exposed to bortezomib for the indicated times. Expression levels of cleaved PARP, cleaved caspase-3, and β-actin (loading control) were evaluated by western blot analyses. BTZ, bortezomib.

**Figure 7:**
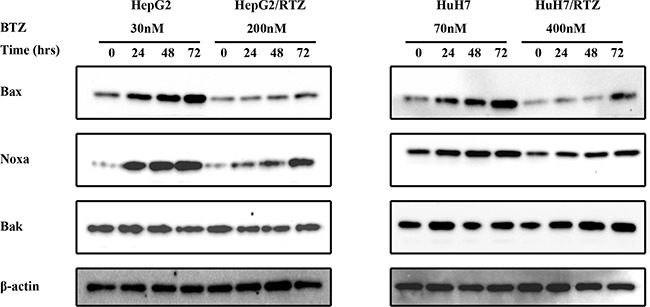
The time-dependent analysis of Bcl-2 family proteins in bortezomib-resistant HCC cells and their parental cells Wild-type HepG2, HepG2/BTZ, wild-type HuH7, and HuH7/BTZ cells were exposed to bortezomib for the indicated times. Expression levels of Bax, Noxa, Bak, and β-actin (loading control) were evaluated by western blot analyses. BTZ, bortezomib.

Bax, Noxa and other Bcl-2 proteins are involved in regulation of apoptosis signal induced by bortezomib. Therefore we detected any differences in the expression of these proteins in bortezomib-resistant HCC cells and wild-type cells. We investigated alterations in the expression of apoptosis-associated proteins in bortezomib-resistant HCC cells and wild-type cells in the presence of various bortezomib concentrations for 48 h. Data illustrated in Figure 6 indicate that bortezomib caused accumulation of Bax and Noxa in all wild-type cells in a dose-dependent manner, starting at a concentration of 10 to 70 nM. Compared with wild-type cells, Bax and Noxa proteins failed to accumulate in response to bortezomib. Additionally, we performed a time-dependent analysis of the expression of apoptosis-associated proteins in bortezomib-resistant HCC cells and wild-type cells. Figure 7 reveals that bortezomib induced a time-dependent increase in the levels of Bax and Noxa in all wild-type cell lines. The maximum effect was observed at 48 h of treatment. However, compared with wild-type cells, Bax and Noxa proteins failed to accumulate in response to bortezomib in bortezomib-resistant HCC cells. These findings suggested that bortezomib up-regulated pro-apoptotic proteins Bax and Noxa in dose- and time-dependent manners in HCC cells. However, the loss of the ability to stabilize and accumulate pro-apoptotic proteins in bortezomib-resistant HCC cells suggested that it caused the acquired resistance to apoptosis.

## DISCUSSION

Most of the information on bortezomib resistance has been obtained from studies that analyzed individual bortezomib resistances in leukemia and lymphoma cell lines. However, there are no reports of bortezomib resistant in liver cancer. This study is the first to establish and characterize bortezomib-resistant HCC cells. We found that (i) resistance to bortezomib can be achieved in a HCC cell line, (ii) the high basal levels of proteasome activities in bortezomib-resistant HCC cells are due to increased expression of proteasome subunits, and (iii) bortezomib-resistant HCC cells acquire resistance to apoptosis by losing the ability to stabilize and accumulate pro-apoptotic proteins.

The ubiquitin–proteasome pathway system regulates many basic cellular processes including cell cycle, division and apoptosis [27, 28]. The proteasome complex is made by a 20s core particle and two 19S regulatory particles. The 20s proteasome is cylindrical and consists of two outer rings called α-subunits and two inner rings called β-subunits. There are three major catalytic activities of proteasomes in the 20s proteasome. They are chymotrypsin-like activity, PGPH activity and trypsin-like activity, corresponding subunits are β5, β1 and β2 [29]. Bortezomib selectively inhibited chymotrypsin-like and PGPH catalytic activities and appeared to affect the β1 and β5 subunits [30]. The direct mechanism of bortezomib resistance has been identified at the proteasome level. Recently studies showed that the direct mechanisms of bortezomib resistance in lymphoma, leukemia and lung cancer cell lines are due to β5 proteasome subunit overexpression and mutations in the β5-subunit. In this study, we found no mutation in the bortezomib-binding pocket of the β5-subunit in HepG2/BTZ and HuH7/BTZ cells. Compared with wild-type cells, chymotrypsin-like and PGPH activities were 3- and 6 -fold higher in HepG2/BTZ cells and PGPH activity was 1.9-fold higher in HuH7/BTZ cells. We also analyzed the expression of the corresponding proteasome subunit. A similar change has been seen in expression level of proteasome. Particularly, expression of the β1 and β5 subunits was increased in the bortezomib-resistant HepG2 cells, whereas an increase in β1 expression was only observed in resistant HuH7 cells. Therefore in bortezomib-resistant HCC cells, the dominant mechanism of resistance at the proteasome level is proteasome subunit over expression. Similar results have been reported recently [31–34]. Kraus et al. reported that bortezomib-adapted HL60 myeloid leukemia cells show increased activity and expression level both of β1 and β5 proteasome subunits [31]. Rückrich et al. indicated that bortezomib-adapted AMO-1, ARH-77, and HL-60 cell lines (myeloma, plasmacytoid lymphoma, and myeloid leukemia, respectively) show increased activity and expression level of the β types of proteasome polypeptides [32]. These increases might partly result from the increased transcription rates of proteasome genes and expression of the respective polypeptides [32]. This notion suggests that the bortezomib resistance is characterized by a reaction pattern including of the upregulation of proteasome gene transcription, expression and activity. Walerych et al. indicated that in cancer cells, p53 missense mutants cooperate with NRF2 to activate proteasome gene transcription, resulting in resistance to the proteasome inhibitor carfilzomib [35]. We did not undertake sequence analysis of *TP53* in bortezomib-resistant HCC cells in this study. Whether the same situation is also present in bortezomib-resistant HCC cells should be confirmed in future experiments.

Several mechanisms of proteasome involvement have been deduced in apoptosis. High expression levels of proteasome have been shown to correlate with apoptosis resistance [36–38]. The key role of the proteasome in the regulation of apoptosis is because of its ability to degrade the regulatory molecules involved in apoptosis. A number of proteasome substrates, including Bax, Noxa, and p53, are critically involved in apoptosis [5, 6, 39–41]. Inhibition of proteasome activity results in the accumulation of these target proteins and induction of apoptosis in many types of tumor cells. In this study, bortezomib-resistant HCC cells acquired resistance to apoptosis as shown by caspase-3 activity as well as caspase-3 and PARP cleavage (Figure 4 and 6). To confirm the cause of resistance to apoptosis in resistant HCC cells, we examined proteasome-targeting proteins in the regulation of apoptosis. We found that the acquired apoptosis resistance in bortezomib-resistant HCC cells was accompanied by loss of the ability to accumulate and stabilize pro-apoptotic proteins such as Bax and Noxa (Figure 5 and Figure 7).

Several Bcl-2 family proteins control the release of some caspase-activating proteins, such as cytochrome *c*, Smac/DIABLO, and HrtA2/Omi into the cytosol. Release of these caspase-activating proteins can be induced by pro-apoptotic members of the Bcl-2 family, such as Bak, Bax, and Bad, but inhibited by anti-apoptotic Bcl-2 family members, such as Bcl-2 and Bcl-XL [42]. Once of the activation of apoptotic signaling, Bax is translocation from cytosol to the organelle membrane, especially the mitochondrial membrane and then permeabilize the mitochondrial outer membrane. As a result, the release of pro-apoptotic factors from mitochondria leads to the activation of caspases. This process defines a direct role of Bax in mediation of apoptotic signaling [43].

Noxa is another pro-apoptotic member of the Bcl-2 protein family [44]. Bax and Bak contain conserved Bcl-2 homology (BH) regions BH1, BH2, and BH3. Noxa is a “BH3-only” type and the most apical regulator of apoptosis. It is activated in response to apoptotic signal and then induces apoptosis [45]. Bax and Noxa are both degraded by ubiquitin-proteasome systems. Treatment with a proteasome inhibitor induces accumulation of Bax and Noxa proteins. In this study, bortezomib caused accumulation of Bax and Noxa in all wild-type HCC cell lines in dose- and time-dependent manners. However, compared with wild-type cells, Bax and Noxa proteins failed to accumulate in response to bortezomib in the bortezomib-resistant HCC cells. Therefore, increased expression of β1 and β5 proteasome subunits caused the failure of Bax and Noxa accumulation in bortezomib-resistant HCC cells and allowed to survive during exposure to bortezomib.

Alterations in the expression of other Bcl-2 family proteins in bortezomib-resistant HCC cells and wild-type cells in the presence of various bortezomib concentrations were not found in this study. The reason may be that these proteins are not correlated by bortezomib in these cells. In addition, several determinants of resistance to bortezomib, such as increased expression level of anti-apoptotic Hsp27 protein [26]. The acquired apoptosis is caused by loss of the ability to stabilize and accumulate p53 protein in bortezomib-resistant Burkitt's lymphoma cells [26]. In this study, we did not find differential expression of Hsp27 and p53 proteins between wild-type and bortezomib-resistant HCC cells. No changing in the expression in all of the BCL-2 family proteins or p53. This means that the function of the mitochondrial pathway—mitochondrial control of apoptosis—is not completely lost in HepG2/RTZ and HuH7/RTZ cells. The DNA damage–p53–mitochondrial pathway–apoptosis cascade was still functional, explaining why HepG2/RTZ and HuH7/RTZ cells are sensitive to doxorubicin (Table 1).

In this study, we established two stable bortezomib-resistant HCC cell lines. There cells display an increase in the expression of proteasome subunits and acquired resistance to apoptosis. After determining the expression of apoptosis related proteins, we found that the ability to accumulate and stabilize Bax and Noxa was lost in these cell lines. Bax and Noxa-induced apoptotic responses were also suppressed. These cell lines will provide useful tools to understand the mechanisms of bortezomib resistance, and to develop of novel therapies for overcome bortezomib resistance.

## MATERIALS AND METHODS

### Chemicals and reagents

Bortezomib was purchased from Selleckchem.com (Selleck Chemicals, Houston, TX, USA), MG-132, MTT (3-[4,5-dimethylthiazol-2-yl]-2,5-diphenyl tetrazolium bromide, were purchased from Sigma-Aldrich (St Louis, MO, USA). The fluorogenic substrates Succ-LLVY-AMC (for proteasome chymotrypsin-like subunit), Ac-RLR-AMC (for proteasome trypsin-like subunit), and Z-LLE-AMC (for proteasome peptidylglutamyl peptide hydrolyzing subunit) and Ac-DEVD-AMC (for caspase-3) were purchased from Enzo Life Sciences (NY, USA).

### Cell culture and development of BTZ-resistant cell lines

The human hepatocellular carcinoma HepG2 cell line was obtained from Riken Cell Bank (Tsukuba, Japan). The hepatocellular carcinoma HuH7 cell lines were obtained from ATCC (USA). All cell lines were cultured in DMEM supplemented with 10% fetal bovine serum (FBS) and 10 μg/ ml. penicillin/streptomycin. Cell cultivation was carried out at 37°C in a humidified atmosphere of 5% carbon dioxide and 95% air. Bortezomib resistant cells were established through a gradual increase in the concentrations of bortezomib for at least 6 months, from 5 nM bortezomib to 1000 nM for HepG2, 40 to 3000 nM for HuH7, thereby yielding resistant cells named HepG2/RTZ and HuH7/RTZ. Bortezomib resistant cells were cultured in bortezomib-free medium for at least 72 h before initiation of experiments to exclude interference of the selective bortezomib concentrations.

### Proteasome activity assay in intact cells

A total of 1×10^5^ cells were cells were seeded in white 96 wells plates. For the investigation of proteasome inhibition, cells were exposed to a range of bortezomib concentrations for 3 h. Wishing twice with cold phosphate-buffered saline containing 5mM MgCl_2_ and 0.2 mg/ml digitonin, which permeabilized the cell membrane without disrupting it. Thereafter, 40 μl of fluorogenic substrate, Suc-LLVY-AMC, w Z-ARR-AMC, and Z-LLE-AMC as added to each well. After incubation for 3 h at 37 °C, fluorescence was measured at 380nm excitation wavelength and 460nm emission wavelength.

### Antibodies, reagents and western blot analysis

Primary antibodies used for western blot analysis were anti-ubiquitin (P4D1) and anti-cleaved caspase-3 (Biolegend, SanDiego, CA, USA), anti-Bax, anti-Noxa, anti cleaved PARP (Santa Cruz Biotechnology, Inc. Santa Cruz, CA, USA), anti-β1 subunit of the 26S proteasome (1:1000) (Abgent, San Diego, CA, anti-β2 subunit and anti-β5 subunit of the 26S proteasome (1:1000) (Bethyl Lab, Montgomery, TX, USA), USA), anti-actin (1:5000) (Abcam, Cambridge, MA, USA). Secondary antibodies (1:2000) were obtained from Dako, (Agilent Technologies, Böblingen, Germany). SDS-PAGE was performed according to Laemmli (1970) [46]. Cultured cells were lysed in lysis buffer. An equal amount of protein extract from each sample was then separated by SDS-PAGE. After electrophoresis, the gels were transferred onto a polyvinylidene difluoride membrane (Hybond-P PVDF Membrane, Amersham Biosciences, Piscataway, NJ, USA) for immunoblotting. The membranes were blocked with 5% dry skim milk in TBST buffer [20mM Tris-HCl (pH7.5), 150mM NaCl, and 0.05% Tween-20] for one hour. After washing in TBST buffer, the membranes were probed with primary antibodies in 1% BSA/TBST buffer for one hour at room temperature. The primary antibody reactions were detected with peroxidase-conjugated goat anti-rabbit or anti-mouse IgG in 1% BSA/TBST buffer, and signal was detected with the Immune-Lite Chemiluminescent assay kit (Bio-Rad Laboratories, Hercules, CA, USA) according to the manufactuer's specifications.

### Preparation of proteasome and enzyme assay

Proteasome extraction from cells was carried out as described previously [47–50]. Briefly, cells (1×10^7^) were harvested, resuspended in 1 ml ATP/DTT lysis buffer [10mM Tris-HCl (pH 8), 5mM ATP, 0.5mM DTT, 5mM MgCl_2_], and incubated on ice for 10 min. This was followed by sonication for 15 s. Lysates were centrifuged at 500 × **g** for 10 min at 4°C. The resulting supernatant containing proteasomes was stable at −80°C for ≥ 1 month with the addition of 20% (v/v) glycerol. Protein concentrations of proteasome extractions from mice and cells were measured using a BCA (bicinchoninic acid) protein assay reagent (Thermo Fisher Scientific Inc. Waltham, MA, USA) with bovine serum albumin as a standard. The fluorogenic substrates Succ-LLVY-AMC, Z-ARR-AMC, and Z-LLE-AMC were used to measure chymotrypsin-like (CT-L), trypsin-like (T-L), and peptidylglutamyl peptide hydrolyzing (PGPH) proteasome activities. Assays were carried out in 50μg, 50mM EDTA and 50 μM fluorogenic substrates in a total volume of 200 μl of ATP/DTT lysis buffer at 37°C°C. The fluorescent rate was determined using a Synergy HT (Bio-TEK Instruments Inc. Winooski, VT, USA) at an excitation wavelength of 395 nm and emission wavelength of 460 nm.

### Cell viability assays and apoptosis assays

Drug toxicity in cells was determined using the MTT. Cells were seeded in 96-wells plates (IWAKI, Tokyo, Japan) at 5000 cells/well. After 24 h enabling attachment, cells were exposed to increasing concentrations of the proteasome inhibitors for 72 h. Thereafter, cells were incubated for 1 h with 100μl of 1 mg/ml MTT solution, followed by addition of 100μl DMSO. Optical density was measured at 540 nm. IC_50_ values were defined as the concentrations that correspond to a reduction of cell growth by 50% when compared to values of untreated control cells and depicted as means's. Cell-free caspase-3 activities were determined by measuring the release of the AMC groups from the caspase-3 specific substrate Ac-Asp-Glu-Val-Asp-AMC. Briefly, followed by preparation of whole cell extracts, the cell extract (30 μg) was then incubated in 100 μl of the assay buffer (50mM Tris-HCI, pH 7.5) with 40 μM of substrate. The reaction mixture was incubated at 37°C for 30 h and the hydrolyzed fluorescent AMC groups were quantified as described above.

### Statistical analysis

Statistical analyses were done with Microsoft Excel software. The Student's *t*-test was applied for independent analysis to evaluate differences between the treatment group and control group.
